# Effect of Feeding Hazelnut Skin on Animal Performance, Milk Quality, and Rumen Fatty Acids in Lactating Ewes

**DOI:** 10.3390/ani10040588

**Published:** 2020-03-31

**Authors:** Adriana Campione, Antonio Natalello, Bernardo Valenti, Giuseppe Luciano, Pablo J. Rufino-Moya, Marcella Avondo, Luciano Morbidini, Camilla Pomente, Barbara Krol, Martyna Wilk, Pawel Migdal, Mariano Pauselli

**Affiliations:** 1 Dipartimento di Scienze Agrarie, Alimentari ed Ambientali, University of Perugia, Borgo XX Giugno 74, 06121 Perugia, Italy; 2 Dipartimento di Agricoltura, Alimentazione e Ambiente, University of Catania, Via Valdisavoia 5, 95123 Catania, Italy; 3 Centro de Investigación y Tecnología Agroalimentaria de Aragón (CITA), Instituto Agroalimentario de Aragón-IA2 (CITA-Universidad de Zaragoza), Avda. Montañana 930, 50059 Zaragoza, Spain; 4 Katedra Żywienia Zwierząt i Paszoznawstwa, University of Environmental and Life Sciences, ul. J. Chełmońskiego 38c, 51-630 Wrocław, Poland; 5 Katedra Higieny Środowiska i Dobrostanu Zwierząt, University of Environmental and Life Sciences, ul. J. Chełmońskiego 38c, 51-630 Wrocław, Poland

**Keywords:** milk, fatty acids, hazelnut skin, by-products, sheep

## Abstract

**Simple Summary:**

The by-products originating from the agro-industry processes are often rich in bioactive compounds. This is the case of hazelnut skin, waste biomass of the pastry industry, rich in polyphenols and fatty acids that, if included in animal feeding, could contribute (i) to improve animal health and products’ quality and (ii) to reduce the feed costs and feed-to-food competition in animal production. We tested this by-product as an ingredient of the diet for lactating ewes. Noteworthy, despite we observed a slight reduction of milk protein percentage, the dietary administration of hazelnut skin improved the composition of the milk lipid fraction by increasing the presence of health-promoting fatty acids and by reducing atherogenic fatty acids. Also, a healthier status of the udder could be hypothesized as a consequence of the inclusion of hazelnut skin in the diet of lactating ewes.

**Abstract:**

The hazelnut skin is waste biomass rich in bioactive compounds that may affect lipid rumen metabolism, ruminant performance, and products’ quality. Therefore, we investigated the effect of dietary hazelnut skin on milk production and composition and on rumen fatty acids in ewes. During 28 days, 20 Comisana lactating ewes received alfalfa hay *ad libitum* plus 800 g/head/day of pelleted concentrate containing 36% dried beet pulp (CTRL group; *n* = 10) or 36% hazelnut skin (HS group; *n* = 10). The protein percentage was lower in HS milk. Milk fatty acids (FA) partially reflected those of rumen content. Total saturated FA (SFA), odd and branched-chain FA, and n-3 polyunsaturated FA (PUFA) were greater in CTRL milk. Total monounsaturated FA (MUFA) and 18:1 trans were greater in HS milk; moreover, HS milk showed a double percentage of oleic acid than the CTRL group. Individual SFA were greater in CTRL milk except for 18:0. Differing from the rumen content, total PUFA, n-6 PUFA, and 18:2 n-6 were comparable between groups. Vaccenic and rumenic acid were greater in HS milk. To conclude, dietary HS slightly reduced milk protein percentage but improved atherogenic index and healthy FA in milk. The content of the somatic cells suggested a healthier udder in the HS group.

## 1. Introduction

The recycling of food processing by-products in animal feeding is an old practice that has been used for centuries, long before the promotion of the 3R principles (reduce-reuse-recycle) to increase the sustainability of the production systems. However, only during the last decades, the research has been focusing on the valorization of agro-industrial wastes as replacers of traditional feedstuffs [1] in order to reduce the environmental impact of livestock productions, especially for ruminants [2]. Including the by-products originating from the agro-industries in animal diets may also enrich their products with biologically active compounds, thus improving the nutritive value and the quality of meat and milk [3,4]. Finally, the use of agro-industrial by-products in animal feeding may be economically advantageous for the industries that could reduce the costs for disposing of the waste biomasses and for the farmers that could attenuate the cost of animal feeding.

In this context, the hazelnut skin (HS) is worthy of attention. It is the perisperm of the hazelnut kernel, and, depending on the food application to which the hazelnut is destined, it is removed during the roasting phase to prevent food bitterness and miscoloration [5], thus representing waste biomass. The HS could be a suitable ingredient in diets for the ruminants because it affords fiber (50–55% dry matter, DM) and energy from fat (15–21% DM), although the protein content is relatively low (6–8% DM) [6]. Moreover, 75% of HS fat is represented by oleic acid, which shows anticholesterolemic activity in humans [7]. However, its application in vivo should be tested, especially considering the high presence of polyphenols, almost exclusively represented by tannins [8]. The effects of tannins on ruminant nutrition are not easily predictable [9]. They may beneficially or adversely affect the feed intake, the availability, and the metabolism of nutrients at rumen level [10], with a consequence on animal performance, health, and product quality [11]. To our knowledge, no study has evaluated the suitability of this by-product as feedstuffs for ruminants. Only our recent paper investigated the sensory and volatile profile of cheese produced with milk from sheep fed HS [12], but the effect on milk yield and quality was not investigated.

In the literature, some agro-industrial by-products have been recently tested as replacers of human-edible feedstuffs (such as cereals) in the diet of farm animals [3,13,14]. However, it could be reasonable to test a by-product to replace another by-product traditionally used in animal nutrition, especially when its production is more environmentally friendly. This could be the case of dried beet pulp that is commonly used to supplement the ruminant diet with fiber and energy since the early 20th century. Mujumadar [15] estimated that 33% of the energy consumption of the sugar factory was destined to beet pulp drying after sugar extraction. Conversely, as HS is discarded immediately after the roasting phase during the hazelnut processing, its water content is low and does not need further stabilization. Both the byproducts show comparable protein and fiber content; thus, HS could be a potential substitute for beet pulp. Moreover, HS may provide bioactive compounds, such as polyphenols and fatty acids, that may improve the quality of ruminant products. Therefore, in the present study, we investigated the effect of replacing dried beet pulp with hazelnut skin in the diet of lactating ewes (i) on the animal performance and milk composition and (ii) on the fatty acid composition of rumen content and milk.

## 2. Materials and Methods

### 2.1. Animals and Diets

The trial was conducted at the Experimental Facility of the Department of Agriculture, Food and Environmental Science, University of Perugia, Italy. Twenty multiparous Comisana lactating ewes at 89 ± 10 days in milk were randomly allotted into two experimental groups (*n* = 10), namely control (CTRL) and hazelnut skin (HS), balanced for parity (2.3 vs. 2.5), current milk yield (824 vs. 804 g/d), and body weight (62.6 vs. 64.0 kg). Within each group, the animals were housed into three different sawdust bedded pens (with two pens hosting 3 animals and one pen hosting 4 animals) where water was always available. Following a 14-day period of adaptation to the experimental diets, the feeding trial lasted 28 days. The experimental diets were formulated to cover the nutrient requirements of a ewe weighing 68 kg and producing 1 kg of milk at 6.5% fat [16]. Diets were composed of chopped alfalfa hay (particle size >4 cm in length), offered ad libitum, and 800 g DM of a pelleted concentrate containing 360 g/kg DM of dried beet pulp (CTRL concentrate) or 360 g/kg DM hazelnut skin (HS concentrate) to replace dried beet pulp. All the concentrate ingredients were incorporated into 5 mm pellets using a pelleting machine (CMS-IEM—Colognola ai Colli, Verona, Italy), operating at 50 °C. The experimental concentrates were offered individually (400 g DM) during each of the two daily milkings (at 7.30 a.m. and 5.30 p.m.) and were completely consumed by all the animals. The ingredients of the experimental concentrates and the chemical composition of feedstuffs are reported in Table 1. The individual milk yield was recorded weekly.

The animal and feeding management adopted reflects the common practice used in a commercial sheep farm-oriented to dairy production. The animals were handled by skilled personnel and in accordance with the European legislation on the protection of animals used for scientific purposes (Directive 2010/63/EU).

### 2.2. Sampling and Analyses

#### 2.2.1. Feed Sampling and Analysis

Offered hay and orts were weighed each day. Weekly, hay and experimental concentrates were sampled, vacuum packed, and stored at –30 °C until analysis. Feed samples were freeze-dried (Minifast D2000 Edwards) and then ground for chemical analysis by mill Cyclotec 1093 (PBI International) using a 1-mm mesh size. Ether extract, ash, and crude protein (CP) were determined according to the AOAC methods [18]. The CP was partitioned into 5 fractions, according to the Cornell Net Carbohydrate and Protein System, as modified by Licitra et al. [17]. Neutral detergent fiber (NDF), acid detergent fiber (ADF), and acid detergent lignin (ADL) were determined according to Van Soest et al. [19] using heat-stable amylase and sodium sulfite, and results were expressed inclusive of residual ash.

The lipid of feed samples was extracted according to Folch et al. [20], and then fatty acid methyl esters (FAME) were obtained according to Christie [21], using C19:0 (Sigma-Aldrich, St. Louis, MO, USA) as an internal standard. The gas-chromatography procedure applied to identify and quantify individual FAME was the same described below for the analysis of milk fatty acids.

Total extractable phenolic compounds and tannins in feeds were analyzed following the procedure originally described by Makkar et al. [22], with modifications as follows. In a 50 mL centrifuge tube, 10 mL acetone 70% (*v*/*v*) was added to 200 mg of finely ground feeds. Samples were vortex-mixed for 1 min and sonicated in a cold water bath (4 °C) for 15 min. Following centrifugation, the supernatant was collected, and the residual solid pellet was re-extracted exactly as above using methanol 80% (*v*/*v*). The supernatants were combined, solvents were evaporated using a rotary evaporator system (Büchi, R-114, Switzerland), and the residue was dissolved in methanol 70% (*v*/*v*). Total phenolic compounds were quantified by mixing 100 μL sample extract, 900 μL distilled water, 500 μL Folin–Ciocalteu reagent (1N), and 2.5 mL sodium carbonate 20% (*w*/*v*). The mixture was vortex-mixed, incubated in the dark for 40 min, and centrifuged at 2500× *g* for 10 min at 4 °C. The absorbance was measured at 725 nm using a double beam UV/VIS spectrophotometer (model UV-1601; Shimadzu Corporation, Milan, Italy). For the analysis of no-tannin phenolics, sample extracts were first treated with insoluble polyvinylpolypyrrolidone (PVPP) in order to remove tannins. Specifically, 2 mL of sample extract (diluted 1:1 with distilled water) was added to 100 mg PVPP, incubated for 20 min at 4 °C, and centrifuged at 2500× *g* for 20 min at 4 °C. The supernatant (200 μL) was mixed with 800 μL of water, and the analysis of no-tannin phenols was performed using Folin–Ciocalteu reagent and 20% sodium carbonate, as previously described. Tannins were calculated as the difference between total phenols and total no-tannin phenols. In all the assays, the quantification of phenolic compounds was achieved using standard solutions of tannic acid (TA) ranging between 0 and 100 μg/mL. Total phenols and tannins were expressed as g TA equivalents/kg dry matter.

#### 2.2.2. Rumen Sample Collection and Analysis of Fatty Acid Profile

On the 21st day of trial, twenty individual rumen content samples were collected before morning feeding using a stomach tube connected to an electrical vacuum pump. Immediately after the collection, the rumen content was frozen at −80 °C, freeze-dried, and stored at –30 °C pending analysis. Freeze-dried rumen content was trans-esterified, as described by Natalello et al. [3]. Briefly, a freeze-dried rumen sample (250 mg) was added in a centrifuge tube containing C19:0 (Sigma-Aldrch, St. Louis, MO, USA) as an internal standard. The basic trans-esterification was performed by the addition of 2 mL of sodium methoxide in methanol (0.5 N, Sigma-Aldrich, St. Louis, MO, USA). The solution was vortexed and left to react for about 10 min at 50 °C. Subsequently, the mixture was cooled at room temperature and added with 3 mL of HCl solution in methanol (3 M, Sigma-Aldrich, St. Louis, MO, USA). The solution was allowed to react for 15 min at 50 °C. Once cooled, 2 mL of a 6% solution of K_2_CO_3_ was added, followed by the addition of 2 mL of hexane. The solution was vortexed, centrifuged, and, finally, the supernatant phase was collected and transferred to another tube. The extraction step was repeated twice. Lastly, the solvent was evaporated, and the FAME extract was resuspended in 0.5 mL hexane. The FAME profile was obtained by gas-chromatography using a Trace Thermo Finnigan GC equipped with a flame ionization detector (ThermoQuest, Milan, Italy) and a 100-m high-polar fused silica capillary column (25 mm i.d., 0.25-μm film thickness; SP 24056; Supelco Inc., Bellefonte, PA). Helium was the carrier gas at a constant flow of 1 mL/min. Total FAME profile in a 1 μL sample volume at a split ratio of 1:50 was determined using the GC conditions reported by Valenti et al. [4]. Specifically, the oven temperature was programmed at 50 °C and held for 4 min, then increased to 120 °C at 10 °C/min, held for 1 min, then increased up to 180 °C at 5 °C/min, held for 18 min, then increased up to 200 °C at 2 °C/min, held for 15 min, and then increased up to 230 °C at 2 °C/min, held for 19 min. The injector and detector temperatures were at 270 °C and 300 °C, respectively. Individual FAME identification was based on a commercial mixture of standard FAME (Nu-Chek Prep Inc., Elysian, MN, USA), individual standard FAME (Larodan Fine Chemicals, Malmo, Sweden). Fatty acids were expressed as g/100 g of total fatty acids.

#### 2.2.3. Milk Sampling and Analyses

Twenty individual milk samples, consisting of proportional volumes of the morning and afternoon milking, were collected weekly and subdivided into aliquots for analyses. One of the two milk subsamples was analyzed immediately after the collection for fat, lactose, protein, casein content (Milkoscan 6000 FT supplied by Foss Electric, Hillerod Denmark), and total somatic cell count (SCC) according to FIL-IDF (1995), using a Fossomatic 5000 (Foss Electric) and expressed as Linear Score (LS = log2 [SCC/12500]). The second aliquot was stored at –80 °C pending analysis for milk fatty acid profile performed as follows. Milk was thawed at 4 °C overnight, and the fat was extracted by progressive centrifugations, as reported by Luna et al. [23], and the FAME were prepared by Christie’s alkaline-catalyzed transmethylation procedure [21], with nonanoic (C9:0) and nonadecanoic (C19:0) acids (Sigma-Aldrich, St. Louis, MO, USA) as internal standards. Individual FAME were determined using the same procedure described above for the fatty acids of rumen content, with the only difference of the split ratio of the carrier gas (1:80 instead of 1:50).

Individual milk protein fractions were analyzed by capillary zone electrophoresis, as reported by Valenti et al. [4]. Individual samples were mixed with a sample buffer (1:2, v:v) composed of 167 mM hydroxymethyl-aminomethane, 42 mM 3-morpholinopropanesulphonic acid, 67 mM ethylenediamine-tetraacetic acid disodium salt dihydrate, 17 mM d,l-dithiothreitol, 6 M urea, and 0.05% (*w*/*w*) hydroxypropylmethylcellulose (HPMC). After 1 h incubation in the dark at room temperature, the samples were centrifuged at 5000× *g* for 5 min, and the top layer fat was removed. Without further preparation, the samples were injected by pressure (20 s at 0.5 psi) for protein separation using a Beckman P/ACE MDQ capillary electrophoresis system controlled by 32 Karat software, version 8.0 (Beckman Instruments, Fullerton, CA, USA), equipped with a UV detector set at 214 nm and an uncoated fused silica capillary (47 cm effective length, 50 µm i.d., 375 µm O.D., slit opening 100 × 800 µm, Beckman Instruments, Fullerton, CA, USA). The separation buffer (pH 3.0 ± 0.1) was 0.19 M citric acid, 20 mM sodium citrate, 6 M urea, and 0.05% (*w*/*w*) HPMC. Electrophoresis runs were carried out at 45 °C with a linear voltage gradient from 0 to 25 kV in 3 min, followed by a constant voltage at 25 kV. Individual proteins were quantified, as reported by Valenti et al. [24], and expressed as a relative percentage.

### 2.3. Statistical Analysis

Data on milk yield, chemical composition, individual proteins, and fatty acid composition were analyzed for repeated measures. The mixed model tested the effect of dietary treatment, time of sampling as fixed factors, and their interaction. The individual animal nested within dietary treatment was considered as a random factor. The pre-treatment data of each parameter was used as a covariate. When the covariate was not significant (*p* > 0.05), it was excluded from the model. Dry matter intake (DMI) intake was analyzed using the model previously described for the milk parameters, but the pen was included as a random factor. Tukey’s adjustment was used for the multiple comparisons of the means. Data on the fatty acid composition of rumen content was analyzed by one-way ANOVA, using the dietary treatment as the fixed factor. Significance was declared when (*p* ≤ 0.05), and the analyses were performed using the statistical software Minitab, version 19 (Minitab, Inc., State College, PA, USA).

## 3. Results

### 3.1. Feed Composition

The chemical composition of the feedstuffs is reported in Table 1. The hazelnut skin used in the present study had a limited content of protein (78.6 g/kg DM), 53.3 g/kg DM of which represented by unavailable nitrogen (C fraction). In contrast, hazelnut skin was high in NDF (511 g/kg DM) and ether extract (226 g/kg DM), the latter being characterized by the high content of oleic acid. Also, hazelnut skin was rich in phenolic compounds (132 g/kg DM), with tannins representing 76.7 g of the total extractable phenols. As a consequence of the inclusion of 360 g/kg hazelnut skin to replace beet pulp, the HS concentrate showed a greater amount of NDF (358 vs. 302 g/kg DM), ADF (226 vs. 135 g/kg DM), ADL (75.6 vs. 15.0 g/kg DM), C fraction of crude protein (29.7 vs. 6.10 g/kg DM), and ether extract (91.5 vs. 16.3 g/kg DM) in comparison with the CTRL. Regarding fatty acids, the HS inclusion increased the percentage of stearic (18:0; 2.40% vs. 1.97%) and oleic acid (18:1 c9, 63.5% vs. 15.9%) in HS concentrate, while the CTRL concentrate was proportionally richer in palmitic (16:0, 22.2% vs. 0.39%), linoleic (18:2 n-6, 52.4% vs. 21.2%), and linolenic acid (18:3 n-3, 5.17% vs. 1.11%). Lastly, HS concentrate had a 20-fold greater content of total extractable phenols than CTRL concentrate (48.2 vs. 2.41 g/kg DM).

### 3.2. Milk Yield and Composition

Table 2 reports the effect of the dietary treatment on milk yield and composition. Daily production (g/d) of milk, fat, protein, lactose, and urea was comparable (*p* > 0.05) between groups. Similarly, lactose percentage and the urea concentration were not affected by the dietary treatment. Milk protein percentage and somatic cell count expressed as linear scores were greater in the CTRL milk (*p* = 0.045 and *p* = 0.024, respectively), while the fat percentage tended to be greater in the HS milk (*p* = 0.082). The percentage of individual milk proteins was not affected by the dietary treatment, with the only exception of the lower percentage of *α*-lactoalbumin found in the milk from ewes fed with HS concentrate (*p* = 0.007).

### 3.3. Fatty Acid Composition of Rumen Content

Most of the fatty acids of the rumen content were significantly different (*p* ≤ 0.05) between the two experimental groups (Table 3). The sum of saturated fatty acids (SFA) was greater in the HS group. This difference was exclusively due to 18:0, found at a double percentage in the rumen content of HS in comparison with CTRL (*p* < 0.001), while 12:0, 14:0, and 16:0 were higher or tended to be higher in the CTRL rumen content. Similarly, total monounsaturated fatty acids (MUFA) were found at a greater percentage (*p* < 0.001) in the rumen content of the animals receiving the HS concentrate. As regards individual MUFA, all the 18-carbon MUFA were greater in the rumen of HS group, except for 18:1 t11 (vaccenic acid, VA), 18:1 c11, and 18:1 c13 that did not differ between groups (*p* > 0.05) and 18:1 c12, greater in the CTRL than HS group (*p* < 0.001). The dietary administration of HS decreased the polyunsaturated fatty acids (PUFA) in the rumen content (*p* < 0.001). In particular, the percentage of 18:2 c9t11 (rumenic acid, RA), 18:2 n-6, and 18:3 n-3 significantly decreased (*p* < 0.001) in the rumen content of HS ewes, and none of the other identified PUFA was greater in the HS than in CTRL rumen. Consequently, the sum of both n-3 and n-6 PUFA was higher in the CTRL group. Lastly, the total odd and branched-chain fatty acids (OBCFA), as well as all the percentage of the individual OBCFA, was greater in the rumen content of the CTRL group (*p* < 0.001).

### 3.4. Milk Fatty Acids

Table 4 reports the effect of the inclusion of hazelnut skin in the diet of lactating ewes on milk fatty acids. The fatty acid composition of milk partially reflected the results described for the rumen content. In particular, similarly to the rumen content, the percentage of total SFA, individual and total OBCFA, and n-3 fatty acids was greater in the CTRL milk (*p* < 0.001), while total MUFA and 18:1 trans were greater in the HS milk (*p* < 0.001). Among SFA, only 18:0 was greater in the HS milk, while 6:0 to 16:0 were greater in the CTRL milk. Regarding the MUFA, with the only exception of 18:1 c11, all the other 18-carbon MUFA were greater in the HS group. Differing from the rumen content, total PUFA and n-6 PUFA did not differ between groups (*p* > 0.05). These results were mainly due to the lack of difference reported for 18:2 n-6 between the milk of the two groups (*p* = 0.767). Finally, vaccenic and rumenic acid in milk showed a different trend in comparison with rumen content, being significantly greater in the HS milk than CTRL milk (*p* < 0.001).

## 4. Discussion

This was the first study investigating the effect of the dietary administration of hazelnut skin on milk yield and composition and on the fatty acid profile of rumen content in lactating ewes. Specifically, 360 g/kg DM hazelnut skin replaced the dehydrated beet pulp in a pelleted concentrate fed to the lactating ewes.

Literature reports that including by-products similar to HS, such as almond hulls, in the diet of ruminants may depress DMI and milk and protein yield [25,26]. This seems to be related to the low content and the poor quality of the protein of almond hulls. Indeed, the fortification of the diet with a nitrogen source may prevent the negative effects on milk production and composition when almonds hull replaces higher-protein feeds [27,28]. We did not observe any effect of dietary HS on DMI and milk yield. Similar to almond hulls, the crude protein of hazelnut skin used in our trial was rich in fraction C, which is linked to the lignin and the Maillard products originated during the hazelnut roasting process. The C fraction of the dietary protein is assumed to be insoluble and undegradable in the rumen and consequently not able to provide available amino acids in the small intestine [18]. However, it should be considered that in our trial, HS replaced dehydrated beet pulp, which is known to provide low digestible protein and needs to be associated with rumen fermentable protein to optimize its nutritive value. Moreover, in both the experimental concentrates, the main protein source was represented by soybean meal, which provides high-quality protein.

Cheese production represents the principal destination of sheep milk. The cheese-making attitude of milk is positively related to total solids. In the present study, the daily production of milk components was not affected by dietary treatments. Conversely, protein percentage that principally affects cheese yield was greater in the CTRL milk. The rumen protein metabolism greatly affects the milk protein. In particular, the rumen microbes preferentially use energy from soluble carbohydrates to support their protein metabolism, and the synchronization between fermentable carbohydrates and soluble protein may enhance the biosynthesis of ruminal protein [29]. The concentration of CP was similar between HS and CTRL, as well as the content of its soluble fraction. Conversely, the C fraction of the protein represented 18.3% and 3.87% of CP in HS and CTRL concentrate, respectively. Moreover, the higher lignin and lower non-fiber carbohydrate (NFC) content in the HS concentrate could have determined a less favorable ratio between soluble protein and NFC in HS fed group, probably justifying the lower milk protein percentage found in the HS milk. Indeed, 30% of the fiber fraction of beet pulp is represented by pectins [30], which can be rapidly fermented to produce energy, while lignin was the predominant fiber fraction in HS. The administration of a diet with an unfavorable energy-to-protein ratio usually determines an increase of ammonia concentration in the rumen. In the present study, we did not investigate the rumen fermentation parameters. As an alternative, the milk urea concentration can give useful indications on the rumen functionality. Indeed, in low energy conditions, the excess of ammonia produced by the degradation of dietary protein is absorbed into the bloodstream and excreted in the form of urea with milk, urine, and feces instead of being converted into new synthesized microbial protein [31]. Therefore, the lack of difference in the urea concentration between HS and CTRL milk seemed to suggest that microbial metabolism was lowered by the dietary administration of HS. This hypothesis seemed to be supported also by the lower percentage of OBCFA observed in the rumen of the HS group. The OBCFA in the rumen and in the ruminant products has been proposed as a potential biomarker for rumen microbial activity. As reviewed by Vlaeminck et al. [32], these fatty acids are synthesized almost exclusively by the rumen bacteria and incorporated in their cell membranes. Therefore, a variation in OBCFA usually reflects changes in the rumen environment, microbial population, and metabolism. Despite the energy supply, we could not exclude that the impairing of ruminal protein metabolism was partially related to the greater intake of polyphenols, especially tannins, by HS ewes. Tannins are known for their antimicrobial activity and for the ability to complex dietary protein, which may prevent the aggression by microbial enzymes while increasing the outflow of by-pass protein and essential amino acids from the rumen to the abomasum and duodenum [33]. This is consistent with Niderkorn et al. [34], who reported that, under in vitro conditions, the inclusion of hazelnut peel in a basal diet for ruminants lowered rumen fermentability and protein degradability. Also, polyphenols possess a strong antioxidant and anti-inflammatory activity that may have played a role in lowering the linear score in HS milk. Indeed, phenols and derivatives of their digestion might be absorbed in the small intestine and reach the tissues for a direct effect, while other phenolic compounds may act indirectly by a general improvement of the health status of the animal [11]. Several reports have shown that in ruminants, polyphenols-enriched diets improve udder status, reduce somatic cells count, and improve the antioxidant status of the animals [35,36]. In agreement with this, in the present study, we observed a reduction of somatic cells. Moreover, as reviewed by Alasavar and Bolling [37], hazelnut may serve as an excellent source of tocopherols. Therefore, it could be speculated that the vitamin E contained in the HS contributed to improving the health status of the ewes receiving the HS concentrate.

The diet is a strategy to manipulate the fatty acid composition of animal products. Usually, this approach is less effective in ruminants than in monogastric because dietary unsaturated fatty acids are largely modified by rumen microbiota metabolism before being absorbed and accumulated in the tissues or in milk [38]. Specifically, dietary unsaturated fatty acids (UFA) undergo a progressive saturation by the enzymes of rumen microorganisms in order to reduce their toxicity. The reason for the sensitivity of microorganisms to PUFA is still unclear; however, it has been demonstrated that the toxicity of UFA increases along with the unsaturation degree [39]. For this reason, dietary linoleic and linolenic acid are the main substrates of the biohydrogenation (BH) process and are extensively converted to stearic acid by the microbial enzymes. In the present study, we observed a greater percentage of 18:0 and total SFA in the rumen of HS ewes, while 18:2 n-6, 18:3 n-3, and total PUFA were lower as compared to CTRL group. Therefore, our data seemed to indicate that the BH proceeded faster or more completely in the HS group. However, it should be considered that the fatty acid composition of the two experimental concentrates differed for both the substrates and the end-product of the BH. In particular, HS concentrate was richer in 18:0, whereas contained less 18:2 n-6 and 18:3 n-3 compared to the CTRL concentrate. In light of this, the fatty acids in rumen content seemed to reflect the fatty acid composition of the diet rather than indicating differences in the BH rate of dietary PUFA. During the BH, several intermediates compounds are produced [38], among which vaccenic and rumenic acid are the most abundant [40]. Rumenic acid (18:2 c9 t11) is produced from linoleic acid via the isomerization of the cis-12 double bond to trans-11, while the synthesis of vaccenic acid (18:1 t11) is a common step along the BH pathway of linoleic and linolenic acid. Taking into account the fatty acid profile of the CTRL diet, the greater percentage of RA in the rumen content of CTRL ewes could have been an expected result. A similar finding could have been expected also for VA. However, it was found at a comparable percentage in the rumen of the two groups. This result might be partially due to the presence of oleic acid as the predominant substrate susceptible to be biohydrogenated in the diet of HS ewes. Similar to PUFA, the oleic acid is extensively biohydrogenated in the rumen. Beam et al. 2000 [41] reported that up to 80% of dietary oleic acid might be lost from mouth to duodenum, mainly due to rumen modifications. Oleic acid is biohydrogenated to 18:0 following a pathway that greatly differs from PUFA. In the past, a direct conversion to stearic acid with no formation of intermediates was hypothesized. However, later studies have shown that the 18:1 c9 is converted to t6÷t16 18:1 [42,43] and that the trans monoenes may be further converted to other trans isomers [44]. Moreover, the dietary administration of oleic acid to dairy cows may increase trans 18:1 fatty acids in the rumen and in milk [45]. Similar to these findings, we observed that the rumen content of HS ewes was characterized by a greater percentage of total trans 18:1. Furthermore, it is well established that in stress conditions, rumen microorganisms can incorporate trans fatty acids into their membranes to change membrane fluidity and maintain its integrity [46]. Therefore, this microbial adaptive response might be caused by environmental stress due to tannins or the excessive presence of dietary lipids. As regard VA, it is supposable that the biohydrogenation of differently available precursors (i.e., linoleic and linolenic acid in the CTRL diet and oleic acid in HS diet) resulted in a comparable production of VA. Also, an impairment of the BH due to the tannins in HS could be hypothesized [11]. In particular, the inhibition of the last step of BH might increase the proportion of VA in the rumen content. The ratio stearic acid/(VA+stearic acid) could be used as an index to estimate the conversion rate of VA to stearate. The lack of numerically or statistically difference between groups for this ratio (data not shown) seemed to rule out the inhibition of the last step of the biohydrogenation in the HS rumen.

The synthesis of VA in the rumen may have an important consequence on the nutritive value of milk, as this fatty acid has been reported as good for human health [47]. Moreover, 18:1 t11 is extensively converted to 18:2 c9t11 by the action of delta-9 desaturase in the mammary gland, with this biosynthetic process being the main source of RA that can be found in the milk [48]. A large number of studies have demonstrated the biological effects of RA against cancer, atherosclerosis, diabetes, and obesity in humans both in vitro and in vivo [49]. Therefore, dietary strategies should aim at maximizing the synthesis of VA in the rumen in order to increase the RA content in milk. Considering that the vaccenic acid is produced exclusively during the BH, it is unexpected that, in the present study, the percentage of VA and RA was greater in milk from HS ewes. However, it should be underlined that the results of rumen fatty acid composition derived from a single sampling (before concentrate administration), while the metabolism of dietary fatty acids is a continuous process. Therefore, it cannot be excluded that the real efflux of fatty acids from the rumen partially differs from that reported in Table 3. Also, milk fat secretion is regulated by complex mechanisms that involve the availability of circulating fatty acids, post-absorption modification, milk fat fluidity, etc. [50,51]. As, in our study, the concentration of total fatty acids was greater in the HS rumen, it could be speculated that the different availability of fatty acids, after the absorption in the gut, could have affected their extraction in the milk.

As regards the other fatty acids, the milk profile partially confirmed the difference observed in the rumen between groups. The sum of MUFA was double both in the rumen and in the milk of HS ewes. Most of the individual MUFA were greater in HS milk (except for 12:1 c9, 14:1 c9, 16:1 c9, and 20:1 t11), but the magnitude of this result was mainly due to the oleic acid, found at a double percentage in the HS than in CTRL milk. The oleic acid in milk arises directly from the diet, or it can be endogenously produced in the mammary gland by the action of delta-9 desaturase from 18:0 [43]. Interestingly, the HS diet afforded a greater quantity of these fatty acids, which were found at a greater level, also, in the rumen of the HS group. Thus, it could be supposed that the mammary gland of HS ewes had greater availability of both 18:1 c9 to be transferred as such to the milk and 18:0 to be used as a substrate for the delta-9 desaturase enzyme. The administration of high-fat diets, especially when characterized by the predominance of long-chain fatty acids (≥18-carbon atoms), negatively affect the de novo synthesis of fatty acids in the mammary gland due to the inhibition of the genes responsible for the endogenous synthesis [52]. As an effect, short and medium-chain fatty acids (≤14-carbon atoms) in milk are usually decreased, which is the result that we observed in the HS group. In our trial, both the diets had a high percentage of long-chain fatty acids, but the fat level in the HS diet was 5-fold greater than in CTRL diet, which could have further enhanced the inhibitory effect of long-chain fatty acids against de novo synthesis in the mammary gland. Reducing the ingestion of some SFA may be favorable for human health. Indeed, medium-chain fatty acids, especially 12:0 and 14:0, are positively related to the increase in food atherogenic index [53]. Finally, PUFAs are known for their potential anti-inflammatory action that may prevent a series of chronic degenerative diseases [54]. Therefore, there is an always increasing interest in enhancing their concentration in animal products, with particular emphasis on the n-3 rather than the n-6 PUFA. In the present study, total PUFA in milk did not differ between HS and CTRL groups. However, the CTRL milk showed a greater content of 18:3 n-3 and total n-3. Nevertheless, both the milk showed an n-6/n-3 ratio fairly lower than 3, which is recommended for human nutrition.

## 5. Conclusions

Concluding, dietary hazelnut skin did not negatively affect DMI and milk yield, tended to increase the fat, and slightly reduced the protein percentage in milk, suggesting that it could replace dried beet pulp in the commercial formulation for lactating ewes with negligible detrimental effects. Moreover, the milk obtained from the ewes receiving the hazelnut skin showed a lower linear score, which might have important positive consequences on the health status of the udder. The most evident effect of the dietary use of hazelnut skin was observed on the fatty acid composition of the rumen content and milk. In milk, the reduction of atherogenic saturated fatty acids and the increase of health-promoting monounsaturated (oleic and vaccenic acid) and polyunsaturated fatty acids (rumenic acid) might enhance the nutritive quality of milk and its derivatives. Finally, the lower proportion of odd and branched-chain fatty acids observed both in the rumen content and in the milk of the HS group could be related to changes in the metabolism or in the rumen microbial population, which might result in the reduction of methane production and emission. Therefore, the effect of hazelnut skin on the ruminal fermentation and microbiome deserves further investigations.

## Figures and Tables

**Table 1:** Ingredients and chemical and fatty acid composition of the experimental treatments and of the hay and hazelnut skin administered to the ewes.

Item	Hay	Hazelnut Skin	Experimental Concentrates ^1^	
			CTRL	HS
Ingredients (g/kg dry matter)				
Hazelnut skin			-	360
Barley			355	330
Wheat bran			99	97
Soybean meal (44% CP)			141	168
Dried beet pulp			360	-
Molasses			25	25
Calcium carbonate			5	5
Sodium bicarbonate			5	5
Dicalcium phosphate			5	5
Sodium chloride			5	5
Chemical composition (g/kg dry matter)				
Crude protein (CP)	150	78.6	158	163
Ether extract	15.8	226	16.3	91.5
Neutral detergent fiber (NDF)	528	511	302	358
Acid detergent fiber (ADF)	429	388	135	226
Acid detergent lignin (ADL)	95.7	203	15	75.6
NFC ^2^	284	214	490	398
Ash	75.6	24.8	63.9	51.8
NEL ^3^	1.31	1.48	1.49	1.55
Protein fractions ^4^ (g/kg dry matter)				
A	39.1	1.80	21.5	8.20
B1	6.90	3.70	5.90	19
B2	71	18.1	100	73.1
B3	19.3	1.60	24.1	32.7
C	13.7	53.3	6.10	29.7
Fatty acids (g/100 g fatty acids)				
14:0	0.86	0.10	0.23	0.09
16:0	24.6	7.04	22.2	9.39
18:0	4.94	2.59	1.97	2.40
18:1 *c*9	5.58	74.9	15.9	63.5
18:2 *c*9 *c*12	18.4	13.7	52.4	21.2
18:3 *c*9 *c*12 *c*15	31.3	0.21	5.17	1.11
Extractable phenolic compounds (g/kg dry matter)				
Total extractable phenols	7.02	132	2.41	48.2
Total extractable tannins	1.32	76.7	0.56	24.6

^1^ CTRL: Concentrate containing 36% dried beet pulp; HS: concentrate containing 36% hazelnut skin; ^2^ Non fiber carbohydrates = 1000 − (CP − (NDF + NDFIP) − Ether Extract − Ash) were NDFIP, representing the protein fraction linked to NDF; ^3^ NEL: Net energy for lactation expressed as Mcal/kg DM; ^4^ Protein fractions: A = NPN; B1 = buffer-soluble true protein; B2 = buffer-insoluble protein–neutral detergent soluble protein; B3 = neutral detergent insoluble protein–acid detergent insoluble protein; C = acid detergent insoluble protein [17].

**Table 2:** The effect of the dietary treatment on milk yield and composition.

Item	Concentrate ^1^ (Diet)		SEM ^2^	*p*-Value ^3^		
	CTRL	HS		D	T	D × T
Dry matter intake (g/d)	2508	2558	19.5	0.214	<0.001	0.986
Milk yield (g)	756	669	40.4	0.525	0.041	0.902
**Milk Composition**						
Fat (%)	5.69	6.46	0.132	0.082	<0.001	0.344
Fat (g/d)	42.8	41.5	1.950	0.866	0.232	0.525
Protein (%)	5.92	5.52	0.055	0.045	0.219	0.002
Protein (g/d)	44.5	36.5	1.867	0.280	0.055	0.673
Lactose (%)	4.60	4.51	0.026	0.297	<0.001	0.722
Lactose (g/d)	34.5	29.6	1.891	0.468	0.069	0.865
Urea (mg/dL)	58.8	57.0	1.210	0.684	0.647	0.007
Urea (g/d)	447	373	16.09	0.322	0.018	0.627
LS (log10 SCC ^4^ × 1000)	3.54	2.81	0.135	0.024	0.003	0.289
**Protein Fractions (% total protein)**						
*α*-lactalbumin	3.20	2.55	0.076	0.007	0.357	0.798
*β*-lactoglobulin	12.2	12.6	0.140	0.321	0.525	0.698
*α*_s2_-casein	7.50	7.61	0.317	0.897	0.030	0.949
*α*_s1_-casein	33.5	32.8	0.451	0.358	<0.001	0.111
*κ*-casein	5.96	5.60	0.167	0.319	0.058	0.694
*β*-casein	34.1	34.9	0.488	0.438	0.006	0.043

^1^ CTRL: Hay + concentrate containing 36% beet pulp; HS: Hay + concentrate containing 36% hazelnut skin; ^2^ SEM: Standard error of mean; ^3^ *p*-values associated with D: dietary treatment, T: time of sampling and interaction; ^4^ SCC: somatic cell count.

**Table 3:** The effect of the dietary treatment on rumen content’s fatty acids (g/100 g of total fatty acids).

Item	Dietary Treatment ^1^		SEM ^2^	*p*-Value ^3^
	CTRL	HS		
Fatty acids (g/100 g of total fatty acids)				
12:0	0.36	0.23	0.036	0.070
13:0 *iso*	0.06	0.04	0.004	<0.001
13:0	0.13	0.06	0.017	0.029
14:0 *iso*	0.32	0.17	0.024	<0.001
14:0	0.92	0.53	0.084	0.013
14:1*t*9	0.15	0.06	0.013	<0.001
15:0 *iso*	0.67	0.35	0.043	<0.001
15:0 *anteiso*	1.62	0.77	0.113	<0.001
15:0	1.65	1.00	0.095	<0.001
16:0 *iso*	1.10	0.47	0.090	<0.000
C16:0	28.1	19.2	1.260	<0.001
C16:1 cis7	0.41	0.44	0.074	0.817
C16:1 cis9	0.18	0.09	0.013	<0.001
17:0 *iso*	0.76	0.32	0.060	<0.001
17:0 *anteiso*	1.47	0.87	0.096	<0.001
C17:0	0.75	0.43	0.045	<0.001
18:0	23.2	43.2	2.670	<0.001
18:1*t*5	0.05	0.13	0.012	<0.001
18:1*t*6+7+8	0.12	0.84	0.098	<0.001
18:1*t9*	0.10	0.40	0.051	<0.001
18:1*t*10	0.14	0.54	0.057	<0.001
18:1*t*11	5.59	6.01	0.164	0.210
18:1*c*6	0.98	1.57	0.082	<0.001
18:1*c*9	7.50	9.13	0.369	0.021
18:1*c*11	0.61	0.61	0.017	1.000
18:1*c*12	0.37	0.33	0.006	<0.001
18:1*c*13	0.04	0.04	0.003	0.325
18:2*c*9*t*11	2.31	0.80	0.220	<0.001
18:2*t*9*t*12	0.06	0.04	0.008	0.172
18:2*t*8*c*13	0.03	0.02	0.004	0.062
18:2 *t*9*c*13	0.55	0.51	0.044	0.698
18:2 n-6	4.86	2.03	0.382	<0.001
18:3 n-6	0.12	0.04	0.031	0.178
18:2 n-3	1.73	0.97	0.131	<0.001
20:0	0.43	0.38	0.016	0.155
20:t11	0.21	0.11	0.035	0.152
20:c11	0.10	0.05	0.013	0.040
20:2 n-6	0.67	0.35	0.057	0.002
20:4 n-6	0.07	0.04	0.007	0.006
21:0	0.06	0.02	0.011	0.034
22:0	0.70	0.50	0.077	0.187
22:1*t13*	0.05	0.02	0.009	0.242
22:1*c13*	0.01	0.01	0.004	0.973
22:4 n-6	0.07	0.06	0.003	0.588
22:5 n-6	0.03	0.01	0.008	0.163
22:6 n-3	0.03	0.04	0.008	0.519
23:0	0.43	0.33	0.030	0.364
24:0	0.37	0.31	0.030	0.364
∑ SFA ^4^	54.1	64.4	1.380	<0.001
∑ MUFA ^5^	16.6	20.4	0.634	<0.001
∑ PUFA ^6^	10.5	4.91	0.780	<0.001
∑ OBCFA ^7^	9.02	4.83	0.546	<0.001
∑ *t*18:1	6.00	7.92	0.380	<0.001
∑ PUFA n-6	5.82	2.53	0.442	<0.001
∑ PUFA n-3	1.76	1.01	0.130	<0.001

^1^ CTRL: Hay + concentrate containing 36% beet pulp; HS: Hay + concentrate containing 36% hazelnut skin; ^2^ SEM: Standard error of mean; ^3^ *p*-values associated with dietary treatment; ^4^ Saturated fatty acids; ^5^ Monounsaturated fatty acids; ^6^ Polyunsaturated fatty acids; ^7^ Odd and branched-chain fatty acids.

**Table 4:** The effect of the dietary treatment on milk fatty acids (g/100 g of total fatty acids).

Items	Dietary Treatment ^1^		SEM ^2^	*p*-Value ^3^		
	CON	HS		D	T	D × T
Fatty acids (g/100 g of total fatty acids)						
4:0	2.12	2.18	0.051	0.658	0.046	0.390
6:0	2.14	1.59	0.057	<0.001	0.022	0.115
8:0	2.32	1.46	0.082	<0.001	0.006	0.111
10:0	8.38	4.09	0.291	<0.001	0.022	0.057
12:0	5.15	2.33	0.205	<0.001	0.053	0.061
12:1*c*9	0.20	0.09	0.008	<0.001	0.350	0.065
14:0	11.5	7.97	0.293	<0.001	0.405	0.002
14:1*c*9	0.24	0.15	0.025	<0.001	0.332	0.012
15:0 *iso*	0.26	0.19	0.006	<0.001	0.023	0.359
15:0 *anteiso*	0.55	0.37	0.015	<0.001	0.402	0.026
15:0	1.38	0.94	0.037	<0.001	0.002	0.511
16:0	29.9	20.8	0.689	<0.001	0.148	<0.001
16:1*c*9	1.06	0.63	0.033	<0.001	0.051	0.043
17:0 *iso*	0.43	0.35	0.012	0.003	<0.001	0.039
17:0 *anteiso*	0.52	0.37	0.014	<0.001	0.089	0.462
17:0	0.88	0.65	0.006	<0.001	0.528	0.303
17:1*c*9	0.30	0.21	0.009	0.002	0.033	0.020
18:0	5.43	10.9	0.416	<0.001	0.009	0.001
18:1*t*5	0.01	0.07	0.087	<0.001	0.720	0.372
18:1*t*6+*t*7+*t*8	0.16	0.84	0.045	<0.001	0.126	0.230
18:1*t9*	0.22	0.67	0.030	<0.001	0.291	0.426
18:1*t*10	0.28	0.64	0.025	<0.001	0.053	0.498
18:1*t*11	0.74	1.74	0.070	<0.001	0.444	0.768
18:1*c*6	0.16	0.84	0.024	<0.001	0.126	0.230
18:1*c*9	14.1	28.6	0.990	<0.001	0.028	0.001
18:1*c*11	0.35	0.48	0.183	<0.001	0.046	0.277
18:1*c*12	0.23	0.23	0.006	0.949	0.064	0.524
18:1*c*13	0.05	0.10	0.004	<0.001	0.780	0.872
18:1*c*14	0.23	0.33	0.008	<0.001	0.040	0.081
18:2c9*t*11	0.49	0.90	0.028	<0.001	0.034	0.581
18:2 n-6	3.34	3.39	0.062	0.767	<0.001	0.002
18:3 n-6	0.02	0.02	0.005	0.151	0.571	0.816
18:3 n-3	1.67	1.16	0.057	<0.001	<0.001	0.173
20:0	0.23	0.24	0.004	0.106	0.006	0.363
20:1*t*11	0.03	0.01	0.001	<0.001	0.137	0.345
20:1*c*11	0.06	0.08	0.057	<0.001	0.069	0.220
20:2 n-6	0.03	0.01	0.003	<0.001	0.395	0.018
20:3 n-6	0.03	0.02	0.001	0.128	0.705	0.177
20:3 n-3	0.02	0.01	0.004	0.007	0.088	0.838
20:4 n-3	0.18	0.14	0.005	<0.001	0.001	0.738
20:5 n-3	0.09	0.05	0.003	<0.001	0.001	0.685
22:0	0.18	0.15	0.005	0.009	0.371	0.025
22:1*c*13	0.02	0.02	0.002	0.475	<0.001	0.006
22:2 n-6	0.02	0.01	0.002	<0.001	0.360	0.031
22:4 n-6	0.04	0.04	0.001	0.783	0.799	0.058
22:5 n-3	0.13	0.09	0.004	0.001	0.001	0.307
22:6-n-3	0.05	0.05	0.002	0.422	0.093	0.704
24:0	0.08	0.08	0.002	0.562	0.852	0.060
24:1*c*9	0.02	0.01	0.001	0.120	0.579	0.037
∑ SFA ^4^	67.37	51.83	7.260	<0.001	0.164	0.008
∑ MUFA ^5^	18.42	35.76	8.560	<0.001	0.093	0.012
∑ PUFA ^6^	6.13	5.88	0.640	0.607	0.363	0.317
∑ OBCFA ^7^	4.02	2.87	0.611	<0.001	0.008	0.889
∑ *t*18:1	1.42	3.97	1.270	<0.001	0.382	0.573
∑ PUFA n-6	3.48	3.49	0.013	0.872	<0.001	0.001
∑ PUFA n-3	2.16	1.49	0.340	<0.001	<0.001	0.323

^1^ CTRL: Hay + concentrate containing 36% beet pulp; HS: Hay + concentrate containing 36% hazelnut skin; ^2^ SEM: Standard error of mean; ^3^ *p*-values associated with D: dietary treatment, T: time of sampling and interaction; ^4^ Saturated fatty acids; ^5^ Monounsaturated fatty acids; ^6^ Polyunsaturated fatty acids; ^7^ Odd and branched-chain fatty acids.
